# Long term efficacy and safety of GH001 in patients with treatment-resistant depression

**DOI:** 10.1192/j.eurpsy.2026.10582

**Published:** 2026-07-31

**Authors:** E. Vieta, B. T. Baune, N. Cardoner, R. M. Dueñas Herrero, L. Janů, J. R. Kelly, S. J. McInerney, A. Nawka, T. Páleníček, V. Pérez Sola, A. Reif, M. E. Thase, V. Valcheva, W. J. Cubala

**Affiliations:** 1 Hospital Clinic de Barcelona, University of Barcelona; 2 Centre for Biomedical Research in Mental Health (CIBERSAM ISCIII), Barcelona, Spain; 3 Department of Psychiatry, University of Muenster, Muenster, Germany; 4 Hospital Santa Creu i Sant Pau, Mental Health Research Group; 5 Parc Sanitari Sant Joan de Deu Hospital de Dia de Numancia, Barcelona, Spain; 6 A-Shine SRO, Pilsen, Czech Republic; 7 Department of Psychiatry, Tallaght University Hospital, Dublin; 8 Department of Psychiatry, University of Galway, Galway, Ireland; 9 Institut Neuropsychiatrické Péče, Praha; 10 Psyon s.r.o, Prague, Czech Republic; 11 Mental Health Institute, Hospital del Mar, Barcelona, Spain; 12 Department of Psychiatry, Psychosomatic Medicine and Psychotherapy, University Hospital Frankfurt – Goethe University, Frankfurt am Main, Germany; 13 Department of Psychiatry, University of Pennsylvania, Philadelphia, United States; 14 GH Research, Dublin, Ireland; 15 Department of Psychiatry, Medical University of Gdańsk, Gdańsk, Poland

## Abstract

**Introduction:**

Treatment-resistant depression (TRD), a persistent condition impacting up to 30% of patients with major depressive disorder, carries substantial personal and economic burden. There remains an unmet need for therapies that provide both rapid and durable improvements in TRD. Preliminary clinical trials indicate that GH001, a synthetic, inhalable formulation of mebufotenin, has the potential for rapid improvement of depressive symptoms in this population.

**Objectives:**

This trial investigated the efficacy and safety of GH001 in patients with TRD in a Phase 2b randomized double-blind trial with a 6-month follow-up.

**Methods:**

In the double-blind part, patients received an individualized dosing regimen (IDR) of up to three escalating doses of GH001 (6, 12, and 18 mg) or placebo IDR on a single day. During the 6-month open-label extension (OLE), patients received up to five GH001 IDR re-treatments.

**Results:**

Eighty-one patients were enrolled (GH001: n = 40; placebo: n = 41; all entered the OLE). GH001 led to a significantly greater reduction in Montgomery-Åsberg Depression Rating Scale (MADRS) total score at Day 8 (primary endpoint) vs placebo (least squares mean difference: -15.5; *P*<0.0001), with significant effects observed from 2 hours post-dose and onwards. Day 8 remission rates (MADRS ≤10) were 57.5% with GH001 vs 0% with placebo. At 6 months, 73.0% (46/63) of OLE completers were in remission. Patients received a mean of four GH001 treatments; 63.5% (n=40) received 1-4 doses. Most adverse events (AEs) were mild or moderate; one serious treatment-emergent AE in the OLE (Migraine) was unrelated to treatment.

**Conclusions:**

GH001 treatment was well tolerated and produced rapid and significant improvements in depressive symptoms in patients with TRD.

**Disclosure of Interest:**

E. Vieta Grant / Research support from: AB-Biotics, Abbott, AbbVie, Adamed, Biogen, Beckley-Psytech, Biohaven, Boehringer-Ingelheim, Clariane, Compass, Dainippon Sumitomo Pharma, Esteve, Ethypharm, Ferrer, Gedeon Richter, GH Research, Glaxo-Smith Kline, HMNC, Intra-Cellular therapies, Idorsia, Johnson & Johnson, Lundbeck, Luye Pharma, Medincell, Merck, Mitsubishi Tanabe Pharma, Otsuka, Rovi, Sanofi-Aventis, Sunovion, and Takeda, Consultant of: Abbott, AbbVie, Adamed, Alcediag, Angelini, Biogen, Biohaven, Boehringer-Ingelheim, Casen-Recordati, Celon Pharma, Compass, Dainippon Sumitomo Pharma, Esteve, Ethypharm, Ferrer, Gedeon Richter, GH Research, Glaxo-Smith Kline, HMNC, Intra-Cellular therapies, Idorsia, Johnson & Johnson, Lundbeck, Luye Pharma, Medincell, Merck, Mitsubishi Tanabe Pharma, Newraxpharm, Newron, Novartis, Organon, Orion Corporation, Otsuka, Roche, Rovi, Sage, Sanofi-Aventis, Sunovion, Takeda, Teva, and Viatris, Speakers bureau of: Johnson & Johnson, Lundbeck, B. T. Baune Grant / Research support from: AstraZeneca, BMBF (Germany), BMG (Germany), DFG (Germany), ERA PerMed, Fay Fuller Foundation, Horizon Europe (European Union), James & Diana Ramsay Foundation (Adelaide), Johnson & Johnson, Lundbeck, La Marató de TV3, National Health and Medical Research Council (Australia), Sanofi-Synthélabo, and Wellcome Trust (UK), Consultant of: National Health and Medical Research Council (Australia). Advisory boards – Biogen, Boehringer, GH Research, Ingelheim, Janssen-Cilag, LivaNova, Lundbeck, Medscape, Novartis, Otsuka, Teva, and Viatris, Speakers bureau of: Angelini, AstraZeneca, Biogen, BMS, Boehringer Ingelheim, Johnson & Johnson, LivaNova, Lundbeck, Medscape, Novartis, Otsuka, Pfizer, Roche, Servier, Sumitomo Pharma, Sunovion, Takeda, Teva, Viatris, and Wyeth, N. Cardoner Grant / Research support from: Ministry of Health, Ministry of Science and Innovation (CIBERSAM), Strategic Plan for Research and Innovation in Health (PERIS, 2016–2020), Recercaixa, Marató TV3, Consultant of: Angelini, Esteve, Janssen, Lundbeck, Novartis, Pfizer, Viatris, Employee of: Hospital de la Santa Creu i Sant Pau; Universitat Autònoma de Barcelona, Speakers bureau of: Adamed, Elsevier, Exeltis, Janssen, Lundbeck, Pfizer, Servier; medical conferences hosted by Boston, Janssen, Lundbeck, and Pfizer, R. M. Dueñas Herrero Grant / Research support from: COMP 006 Subinvestigator. BPL 201: PI, L. Janů: None Declared, J. R. Kelly Grant / Research support from: Health Research Board (ILP-POR-2022-030, DIFA-2023-005, HRB KTA-2024-002), S. J. McInerney Grant / Research support from: Principal investigator – GH Research and Transcend Therapeutics, Speakers bureau of: Janssen and Lundbeck, A. Nawka Grant / Research support from: Principal Investigator - GH Research, T. Páleníček Shareholder of: Psychedelická klinika s.r.o., Společnost pro podporu neurovědního výzkumu s.r.o., and AVI-X Aviation Experts s.r.o. Founder – PSYRES (Psychedelic Research Foundation), Grant / Research support from: Principal investigator – Compass, GH Research, MAPS, and Ketabon, Consultant of: CB21 Pharma and GH Research, V. Pérez Sola Consultant of: Consultant, honoraria, or grants – AB-Biotics, AstraZeneca, Bristol Myers Squibb, CIBERSAM, FIS-ISCiii, Janssen Cilag, Lundbeck, Medtronic, Otsuka, and Servier, A. Reif Grant / Research support from: Medice and Janssen, Consultant of: Janssen/Johnson & Johnson, Boehringer Ingelheim, Compass, GH Research, SAGE/Biogen, LivaNova, Medice, Shire/Takeda, Newron, MSD, AbbVie, cyclerion and Polpharma, Speakers bureau of: Janssen/Johnson & Johnson, Boehringer Ingelheim, Compass, GH Research, SAGE/Biogen, LivaNova, Medice, Shire/Takeda, Newron, MSD, AbbVie, cyclerion and Polpharma, M. E. Thase Grant / Research support from: Acadia, Alkermes, Axsome, Intra-Cellular Therapies, Janssen, National Institute of Mental Health, Otsuka, Patient-Centered Outcomes Research Institute (PCORI), and Takeda, Consultant of: Autobahn Therapeutics, Axsome, Clexio Biosciences, Gerson Lehrman Group, GH Research, Lundbeck, Janssen, Johnson & Johnson, Luye Pharma, Merck, Object Pharma, Otsuka, Pfizer, Sage, Seelos Therapeutics, Sunovion, and Takeda, V. Valcheva Shareholder of: Stock option holder of GH Research, Employee of: GH Research, W. J. Cubala Grant / Research support from: Acadia, Angelini, Beckley Psytech, GH Research, HMNC Brain Health, Intra-Cellular Therapies, Janssen, MSD, Neumora, Novartis, Otsuka, Recognify Life Sciences, Consultant of: Douglas Pharmaceuticals, GH Research, Janssen, MSD, and Novartis, Speakers bureau of: Angelini, GH Research, Janssen, and Novartis.

